# The Mechanism of the Interfacial Charge and Mass Transfer during Intercalation of Alkali Metal Cations

**DOI:** 10.1002/advs.201600211

**Published:** 2016-09-28

**Authors:** Edgar Ventosa, Bianca Paulitsch, Philipp Marzak, Jeongsik Yun, Florian Schiegg, Thomas Quast, Aliaksandr S. Bandarenka

**Affiliations:** ^1^ Analytical Chemistry—Center for Electrochemical ScienceRuhr‐Universität BochumUniversitätsstr. 15044780BochumGermany; ^2^ Physik‐Department ECSTechnische Universität MünchenJames‐Franck‐Straße 185748GarchingGermany; ^3^ Nanosystems Initiative Munich (NIM)Schellingstraße 480799MunichGermany

**Keywords:** batteries, electrified interfaces, impedance spectroscopy, intercalation, intercalation mechanism

## Abstract

**Intercalation of alkali metal cations**, like Li^+^ or Na^+^, follows the same three‐stage mechanism of the interfacial charge and mass transfer irrespective of the nature of the electrolyte, electrolyte composition or electrode material.

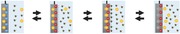

It is nowadays a nonsurprising fact that the solar and wind power noticeably contribute to the energy supply in a number of countries.^1^, ^2^ However, the major drawback of the modern renewable power sources is that they are still very dependent on the natural phenomena fluctuating with time. In order to successfully solve the generation versus consumption problem, efficient energy storage systems (ESSs) are necessary. The energy storage systems can be categorized according to the methods to store it such as chemical, electrical, electrochemical, mechanical, and thermal ESSs. According to the Electric Power Research Institute, the present ESSs are dominated by different pumped‐storage hydroelectricity systems worldwide; however, they have serious geographical limitations to apply in general cases.^3^ Rechargeable batteries, among others, are considered to have a great potential as a viable alternative due to a large number of advantages such as the high energy conversion efficiency, long cycle life, and relative ease to control over the storage and release of energy.^4^, ^5^ However, further optimizations of the existing battery systems are required. For that a deeper understanding of mechanisms governing their performance is necessary.

The performance of the majority of modern batteries is largely determined by the status of the electrode/electrolyte interfaces. Interfaces at battery electrodes have been studied for a long time; however, they are not fully understood yet. For instance, the so‐called solid electrolyte interface (SEI) formed at the negative electrode of common Li‐ion batteries has attracted a special attention since it drastically influences the key parameters in the performance of a battery, e.g., cycle‐life and safety.^6^, ^7^ This SEI is probably the most important and the least understood element in rechargeable Li‐ion batteries.^8^ The lack of deep understanding of it has been attributed to its complexity. However, how good is the current understanding of interfacial mechanisms of mass and charge transfer even in simpler, e.g., aqueous, battery systems? The answer is commonly believed to be “well‐understood”. On the contrary, e.g., a recent study^9^ revealed that the interfacial mechanism at model Na‐intercalation material in aqueous media is likely not as simple. In this work we demonstrate that the interfacial charge and mass transfer in multiple battery systems (though without organic dielectric thin films at the electrode surfaces) involving different Li‐ion, Na‐ion and K‐ion intercalation materials in aqueous and organic electrolytes proceeds through at least three‐stage interfacial mechanism, being similar for all these systems.

In the following, we give a brief rationale for the particular selection of battery systems used in this work. First, the voltage of the state of the art Li‐ion batteries (>3.7 V) requires the use of organic electrolyte solutions with larger electrochemical stability window compared to aqueous systems. However, the state‐of‐the‐art carbonate‐based solutions decompose at the very cathodic potentials necessary to lithiate graphite. The products of the decomposition precipitate onto the electrode and form a protecting film, which is electrically insulating, thus preventing further electrolyte decomposition. Therefore, for this work, two Li intercalation anode materials (Li_4_Ti_5_O_12_ and TiO_2_) were selected, which operate at more positive potentials (1.5–1.8 V vs. Li/Li^+^) without the formation of thick organic films.^10^, ^11^, ^12^ As a common cathode material, LiFePO_4_ operating at low potentials (≈3.55 V vs. Li/Li^+^) and preventing the presence of cathode electrolyte interphase was chosen. For the aqueous systems, state‐of‐the‐art Prussian Blue Analogues (PBAs)^13^, ^14^, ^15^ electrochemically grown as thin films were used. Some of PBAs were also tested in organic electrolytes. PBAs are considered as very promising and relatively cheap electrode materials for future generations of aqueous and nonaqueous Li‐ and Na‐ion battery systems,^9^ and, therefore, were included as the model systems in this work.

In order to elucidate the interfacial processes during quasi‐reversible (de‐)intercalation, it is worth to notice that the process of (de‐)intercalation in many state‐of‐the‐art and perspective electrode materials starts with the change of the oxidation state of the host metal cation. For instance, in the case of numerous PBAs, the oxidation state of Fe is changed between 2+ and 3+. This process is very fast due to a relatively good electronic conductivity of intercalation electrodes. This is also schematically shown in **Figure** **1**A,B. When the oxidation state of the host metal is changed, the resulting excessive charge should be balanced via intercalation or de‐intercalation of the alkali metal cations. However, in intercalation compounds, the mobility of cations is generally the limiting factor for the solid state diffusion. The mobility of cations in the bulk of solid intercalation compounds is significantly lower than that of ions in liquid electrolytes, irrespective of the nature of the solvent (organic or H_2_O‐based). As a result, a sort of “total uncompensated charge” can likely appear (Figure 1B) provoking in the case of de‐intercalation a specific adsorption of anions from the electrolyte side to temporarily (and likely partly) compensate it, as illustrated in Figure 1C. The term “specific adsorption” in this context means that anions go through the first water layer from the electrolyte side and directly interact with the electrode surface. This often causes an additional interfacial charge transfer from negatively charged anions (during de‐intercalation). In the following stage (Figure 1D), all expected alkali metal cations leave the electrode, and anions subsequently tend to desorb. Importantly, in many cases, the stages shown in Figure 1 are quasi‐reversible. Therefore, one can schematically write the following general equations for the above‐described mechanism (it is also described in more detail in Section 3, Supporting Information)

(1) $$AM_{x}M\prime \;[{\mathrm{TML}}_{y}]-\mathrm{1e}-\;↔\;AM_{x}M\prime \;{\left[{\mathrm{TML}}_{y}\right]}^{+}$$

(2) $$AM_{x}M\prime \;{\left[{\mathrm{TML}}_{y}\right]}^{+}\;+\;A^{-}\;↔\;AM_{x}M\prime \;[{\mathrm{TML}}_{y}]\;A$$

(3) $$AM_{x}M\prime \;[{\mathrm{TML}}_{y}]\;A\;↔\;AM^{+}\;+\;A^{-}\;+\;AM_{x-1}M\prime \;[{\mathrm{TML}}_{y}]$$

where TM is a transition metal (e.g., Fe or Ti). **AM**—alkali metal cation (e.g., Li^+^, Na^+^ or K^+^), M′—an “optional” transition metal cation which does not participate in the redox reactions (e.g., Ni or Cu), L*_y_* is a “ligand” (e.g., CN^−^ or O^2‐^), A^−^ is an anion (e.g., NO_3_^−^).

**Figure 1:**
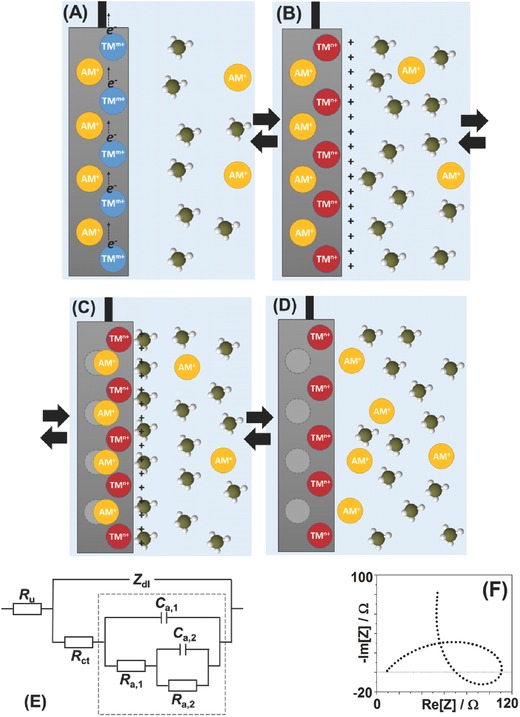
Schematic representation of the suggested stages for the interfacial charge and mass transport during (de‐)intercalation of alkali metal cations (AM^+^). A) Fast oxidation of a transition metal (TM^m+^). B) Appearance of an “uncompensated charge” due to a slow diffusion of the AM^+^ in the solid. C) Specific adsorption of anions as a mean to temporary neutralizes this charge. D) The AM^+^ and anions leave the surface. See Section 3 in the Supporting Information, for further details. E) The equivalent electric circuit (*R*_u_—uncompensated resistance, *Z*_dl_—impedance of the double layer, *R*_ct_—charge transfer resistance, other *R* and *C* elements are adsorption (pseudo)resistances and (pseudo)capacitances, respectively). F) Schematic of the expected Nyquist plot are uniquely revealing three intrinsically connected and quasi‐reversible processes shown in (A–D). See text for further details.

It should be noted that there is unfortunately a very limited number of techniques capable to prove the above mentioned scheme experimentally. However, there is a relatively simple nondestructive mean to do so, namely electrochemical impedance spectroscopy (EIS). This is one of only few suitable approaches, when the system intercalates and de‐intercalates cations during the probing, which likely enables the most adequate characterization.

As related to the EIS‐characterization, the general scheme involving three interconnected quasi‐reversible electrochemical stages where the last stage does not involve the net charge transfer often leads to a number of equations, which can be equivalently represented using a circuit shown in Figure 1E.^16^ Depending on the exact combination of the kinetic constants in those equations at a given potential, the “capacitances” and “resistances” in the selected part of the circuit in Figure 1E might formally be negative. This leads to the unique shape of the impedance spectra at some electrode potentials with the “negative loops”, as shown in Figure 1F. Thus, the appearance of such loops and, most importantly, the ability to fit those spectra to the physical model shown in Figure 1E would confirm the expectations (Section 3, Figures S10–S12 in the Supporting Information for further step‐by‐step explanations related to the modeling and fitting).

**Figure** **2** characterizes thin films of PBAs (Section 1, Figures S1–S7 in the Supporting Information, regarding their electrochemical preparation and characterization) in different aqueous, mixed H_2_O/CH_3_CN, and organic electrolytes.

**Figure 2:**
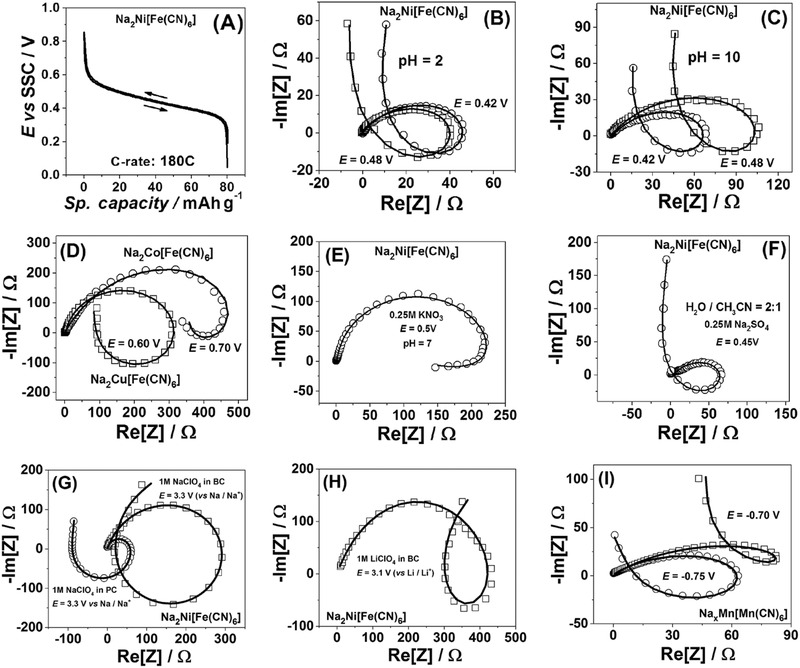
A) A typical potential profile for Na_2_Ni[Fe(CN)_6_] thin films recorded in 0.25 m Na_2_SO_4_ aqueous solutions and examples of their impedance spectra at B) pH = 2 and C) pH = 10. D) Examples of impedance spectra of Na_2_Co[Fe(CN)_6_] and Na_2_Cu[Fe(CN)_6_] in 0.25 m Na_2_SO_4_ aqueous solutions at pH = 7. E,F) Examples of impedance spectra for Na_2_Ni[Fe(CN)_6_] thin films in E) aqueous 0.25 m KNO_3_ and F) 0.25 m Na_2_SO_4_ mixed H_2_O/acetonitrile (2:1 volume ratio) electrolytes. G,H) Examples of impedance spectra of Na_2_Ni[Fe(CN)_6_] in organic electrolytes (PC states propylene carbonate, BC—1,2‐butylene carbonate). Electrolyte compositions are given in the legends. I) An anode material Na*_x_*Mn[Mn(CN)_6_] in an aqueous electrolyte of 10 m NaClO_4_ demonstrate the same features. Open symbols in B–I) are experimental data (corrected for *R*_u_) and solid lines represent the results of the fitting to the model shown in Figure 1E. Frequency range 50 kHz–0.1 Hz/1 Hz.

Figure 2A shows typical galvanostatic (dis‐)charge curves at 180 C for Na_2_Ni[Fe(CN)_6_] electrodes in aqueous Na_2_SO_4_ electrolytes. The curves show only a small hysteresis revealing generally high reversibility of the Na^+^ (de‐)intercalation. As can be seen in Figure 2B–I, within the wide potential ranges all the state‐of‐the‐art PBA‐electrodes in all investigated electrolytes demonstrate well‐defined representative features, similar to those schematically shown in Figure 1F (see more examples, Sections 3 and 4 in the Supporting Information). Moreover, the model representing the three‐stage mechanism (Figure 1E) can fit all the data with low normalized root‐mean‐square deviations (normally below 1%–2%). It should be noted that the Kramers‐Kronig check procedures do not reveal any problems with the spectra (Section 5, Figures S16–S19 for examples in the Supporting Information), additionally suggesting that the loop‐features do not appear as a result of nonlinearity or nonstationarity or other experimental artefacts. Interestingly, the model of the three‐stage mechanism can explain the data for drastically different environments, such as at different pHs (Figure 2B,C), where higher pH values can change the (de‐)intercalation kinetics and Na_2_Ni[Fe(CN)_6_] film stability but not the mechanism itself. The introduction of K^+^ instead of Na^+^ and changing the nature of the anions from SO_4_^2−^ to NO_3_^−^ (Figure 2E), and even the introduction of a significant amount of an organic solvent (acetonitrile, Figure 2F) do not change the interfacial mass and charge transfer scheme, which can still describe the impedance response very well. The same remained true if other PBA‐electrodes, namely Na_2_Co[Fe(CN)_6_] and Na_2_Cu[Fe(CN)_6_] (cathode materials, Figure 2D) or Na*_x_*Mn[Mn(CN)_6_] (anode material, Figure 2I) were used. Moreover, PBA‐electrodes demonstrate similar impedance response in organic, propylene carbonate, and butylene carbonate, electrolytes (Figure 2G,H). Thus, we hypothesize that all PBA battery materials in all aqueous or organic solvents would demonstrate similar three‐stage mechanism for the interfacial mass and charge transfer.

With the aim of extrapolating our findings, we studied the interfacial charge and mass transport in other systems, where the change in the oxidation state of the host metal determines the (de‐)intercalation of alkali metal cations. In the following system, a number of parameters was changed with respect to the PBA‐based systems: (i) solely organic electrolyte solutions, (ii) solely Li^+^ intercalation compounds, (iii) hexafluorophosphate (PF_6_^−^) anions, (iv) porous paste electrodes (Li_4_Ti_5_O_12_, TiO_2_ or C‐LiFePO_4_, carbon black and binder in 76:15:9 wt%) and (v) battery‐type cell (a three‐electrode coaxial cell optimized for EIS measurements^17^). We have chosen TiO_2_ and LiFePO_4_ as intercalation materials because they operate within the electrochemical window of carbonate‐based electrolytes making them excellent model intercalation systems. Commercially available Li_4_Ti_5_O_12_, anatase TiO_2_, and C‐LiFePO_4_ (**Figure** **3**A,C,E) showed the typical electrochemical behavior of these materials.^10^, ^11^ The flat potential plateau seen in the potential profiles of these two materials is due to the phase transformations occurring during (de‐)intercalation. Therefore, EIS spectra were recorded at different states of charge instead of different potentials (Sections 2 and 4, and Figure S13 in the Supporting Information for more details). The EIS spectra of Li_4_Ti_5_O_12_, anatase TiO_2_, and C‐LiFePO_4_ electrodes (Figure 3B,D,F) again demonstrate the characteristic feature associated with three intrinsically connected and quasi‐reversible processes. Note that the multistage interfacial mechanism was revealed at different states of charge for many cycles (Figures S14 and S15, Supporting Information). The fact that (de‐)intercalation in these battery electrodes in organic electrolyte solutions also proceeds through a three‐stage interfacial mass and charge transfer mechanism suggests that it is not specific for a certain battery material in a particular environment/conditions but common for multiple battery systems.

**Figure 3:**
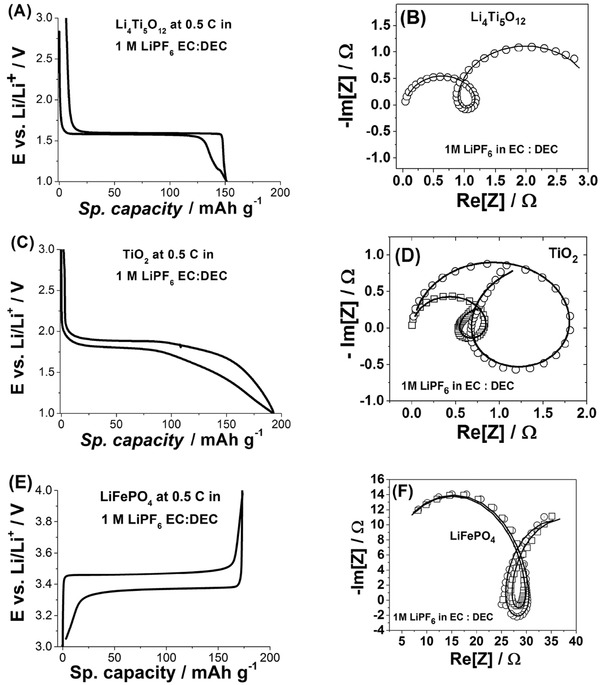
A) A typical potential profile for Li_4_Ti_5_O_12_ electrodes cycled at 0.5 C (87 mA g^−1^) in a standard battery electrolyte (1 m LiPF_6_ in ethyl carbonate (EC): diethyl carbonate (DEC)) and B) an example of its impedance spectra. C) A typical potential profile for anatase TiO_2_ electrodes cycled at 0.5 C (168 mA g^‐1^) in 1 m LiPF_6_ in EC:DEC and D) examples of its impedance spectra. E) A typical potential profile for C‐LiFePO_4_ electrodes cycled at 0.5 C (80 mA g^−1^) in a standard battery electrolyte (1 m LiPF_6_ in EC:DEC) and B) an example of its impedance spectra. (Open symbols in B,D,F) are experimental data (corrected for *R*_u_) and solid lines represent the results of the fitting to the model shown in Figure 1E. The frequency range for EIS spectra was 50 kHz–5 Hz.

In conclusion, we have demonstrated that the three‐stage mechanism^9^ for the interfacial mass and charge transfer occurs during (de‐)intercalation of alkali metal in diverse battery systems. It was revealed in all of the studied cases: aqueous and organic electrolyte solutions, Li^+^, Na^+^, and K^+^ intercalation compounds, SO_4_^2−^, NO_3_^−^, and PF_6_^−^ anions, thin films and porous paste electrodes. This indicates that this mechanism is rather common in batteries, surprisingly for both cathodes and anodes. Therefore, the interpretation of the battery test data as well as the design of the new battery systems should involve the above‐described considerations, especially in the case of fast (de‐)intercalation kinetics. In particular, the commonly observed in battery research “impedance loops” should in many cases be an indication of the specific mechanism of the interfacial charge and mass transfer, rather than an experimental error. However, further investigations including more experimental techniques, for instance other potentiodynamic^18^, ^19^ or electrochemical microscopic^20^, ^21^, ^22^ techniques, and aiming at covering a broader choice of intercalation materials and electrolytes are necessary for deeper understanding of the factors governing intercalation of alkali metal cations.

## Experimental Section

*Electrochemical Measurements in Aqueous Media*: Electrochemical experiments in aqueous media were carried out in different glass cells using a three‐electrode configuration, consisting of the PBA working electrodes, a platinum wire as a counter electrode and an Ag/AgCl (SSC, sat.) reference electrode, to which all the electrode potentials for the aqueous systems were referred. As the substrates for the PBA thin film working electrodes, either a sputtered gold on glass arrandee, an Au electrochemical quartz crystal microbalance (EQCM) electrode (SRS, Stanford, USA) or an Au(111) single crystal (Mateck, Jülich, Germany; polished down to 30 nm) were employed. The PBA thin films were electrochemically deposited from aqueous solutions and characterized as described in Supporting Information, Section 1. Electrochemical impedance spectroscopy measurements were performed as described in detail elsewhere^9^ (see Section 1 in the Supporting Information, for details). The impedance data were analyzed using home‐made software.^23^, ^24^

*Materials and Electrochemical Characterization of Electrodes in Organic Media*: Commercially available Li_4_Ti_5_O_12_ (2 m^2^ g^−1^), anatase TiO_2_ (100 m^2^ g^−1^) and carbon‐coated LiFePO_4_ (2–5 wt% content of carbon) materials were received from MTI Corp (USA), Sachtleben Chemie (Germany) and Phostech Lithium, Clariant Canada Inc. (Canada), respectively. The porous paste electrodes were prepared following the standard procedure (see Section 2 in the Supporting Information, for more details). A current density of 80, 87, and 168 mA g^−1^ (C‐rate of 0.5 C) was applied to C‐LiFePO_4_, Li_4_T_5_O_12_, and TiO_2_ electrodes, respectively, within the potential window 4.0–3.0 V and 3.0–1.0 V for cathode (C‐LiFePO_4_) and anode materials (Li_4_T_5_O_12_ and TiO_2_). All the electrode potentials in organic electrolytes are reported versus Li/Li^+^ (in 1 m Li^+^). All electrochemical experiments were performed using a three‐electrode coaxial cell specially designed for EIS measurements (Sections 2 and 4, and ref. 17 in the Supporting Information) using a Bio‐Logic VMP‐3 potentiostat (Bio‐Logic). A potential perturbation with 10 mV amplitude was applied in the frequency range from 50 kHz to 5 Hz.

## Supporting information

As a service to our authors and readers, this journal provides supporting information supplied by the authors. Such materials are peer reviewed and may be re‐organized for online delivery, but are not copy‐edited or typeset. Technical support issues arising from supporting information (other than missing files) should be addressed to the authors.

Supplementary
