# Supplementary material for: The Mechanism of the Interfacial Charge and Mass Transfer during Intercalation of Alkali Metal Cations
Source: Adv Sci (Weinh). 2016 Sep 28;3(12):1600211. doi: 10.1002/advs.201600211 (PMC5157184; doi:10.1002/advs.201600211)
Supplement: Supplementary file 1 — Supplementary [file ADVS-3-0-s001.pdf]

Copyright WILEY-VCH Verlag GmbH & Co. KGaA, 69469 Weinheim, Germany, 2016.

ADVANCED SCIENCE Open Access

Supporting Information

for *Adv. Sci.,* DOI: 10.1002/advs.201600211

The Mechanism of the Interfacial Charge and Mass Transfer during Intercalation of Alkali Metal Cations

Edgar Ventosa, Bianca Paulitsch, Philipp Marzak, Jeongsik Yun, Florian Schiegg, Thomas Quast, and Aliaksandr S. Bandarenka**

Copyright WILEY-VCH Verlag GmbH & Co. KGaA, 69469 Weinheim, Germany, 2013.

Supporting Information

The Mechanism of the Interfacial Charge and Mass Transfer during Intercalation of Alkali Metal Cations

Edgar Ventosa, Bianca Paulitsch, Philipp Marzak, Jeongsik Yun, Florian Schiegg, Thomas Quast, Aliaksandr S. Bandarenka**

1. PBA film formation and characterization

Cyclic voltammetry was used for cleaning the substrates, deposition of thin films and intercalation experiments. For all cyclic voltammograms, the scan rate was fixed to 50 mV/s. Before the deposition of PBA thin films, the working electrode was electrochemically pretreated by cycling the electrode potential between 0 V and 1.4 V vs SSC in 0.1 M H_2SO_4 (96 %, Merck Suprapur), until the cyclic voltammogram was steady. In between of each process step (cleaning, deposition or (de-)intercalation) the cell was rinsed with ultrapure water (18.2 MΩ, Evoqua, Germany). The fresh deposition solution was de-oxygenated with Ar (5.0, Westfalen AG) for > 15 minutes in the preconditioning cell before the experiments.

For the electrochemical deposition of $Na_2Co[Fe(CN)_6]$ thin films on an arrandee^{TM} substrate, the deposition solution contained 0.25 M Na_2SO_4 (≥99 %, Sigma Aldrich), 5 x 10^{-4} M $K_3Fe(CN)_6$ (99 %, Sigma Aldrich) and 5 x 10^{-4} M $CoCl_2 \cdot 6H_2O$ (98 %, Sigma Aldrich). The cyclic voltammetry was performed from 0.05 V to 0.7 V vs SSC for ~60 cycles (Figure S1A).

For the $Na_2Cu[Fe(CN)_6]$ thin film deposition on an EQCM crystal plated with Au (SRS, Stanford, USA) the solution contained 0.25 M Na_2SO_4, 1.25 x 10^{-4} M $K_3Fe(CN)_6$ and 1.25 x 10^{-4} M $CuCl_2 \cdot 2H_2O$ (≥99 %, Sigma Aldrich). The potential range for the CV-deposition was set from 0.5 V to 0.82 V vs SSC, and ~800 cycles were performed (Figure S1B).

The solution for the $Na_2Ni[Fe(CN)_6]$ deposition contained 0.25 M Na_2SO_4, 5 x 10^{-4} M $K_3Fe(CN)_6$ and 5 x 10^{-4} M $NiCl_2 \cdot 6H_2O$ (99.3 %, Alfa Aesar). The Au(111) single crystal utilized as a working electrode was cycled in a potential range from 0 V to 0.9 V vs SSC using hanging meniscus configuration. For the deposition of the $Na_2Ni[Fe(CN)_6]$ thin film ~60 cycles were carried out (Figure S1C). Care was taken that the final current densities in following depositions of this material were comparable to the other samples.

The $Na_xMn[Mn(CN)_6]$ thin film was deposited on an arrandee™ substrate in a solution of 0.25 M Na_2SO_4, 2 mM $MnSO_4 \cdot H_2O$ (≥98.0%, Amresco) and 2 mM $K_3Mn(CN)_6 \cdot 3H_2O$ (synthesized according to Brauer[1]) at pH=4.7. The vertices for ~100 deposition cycles were chosen as -1.18 V and -0.6 V vs SSC (Figure S2A).

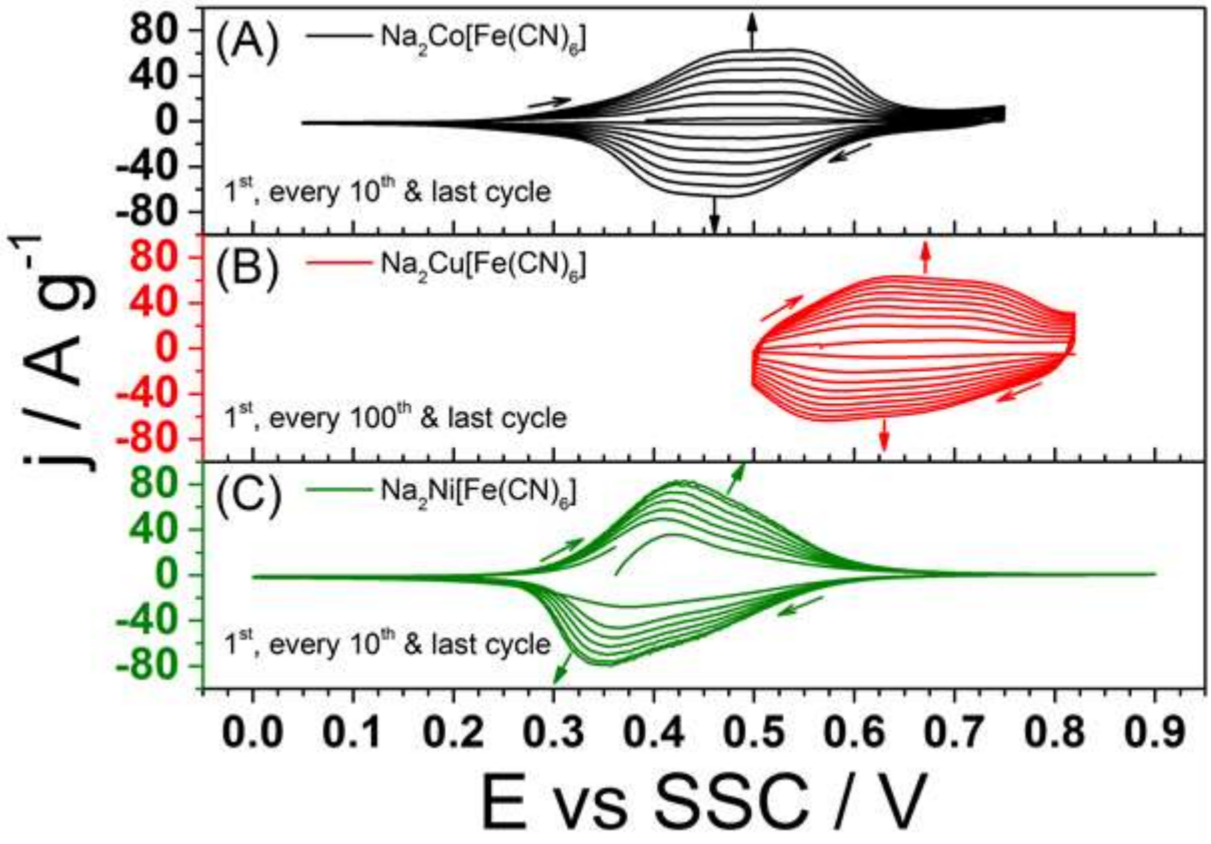

Figure S1. Typical cyclic voltammograms recorded during electrochemical deposition of the PBA thin films. Potential scan rate is 50 mV/s. The current density was normalized by the final mass of the deposited materials.

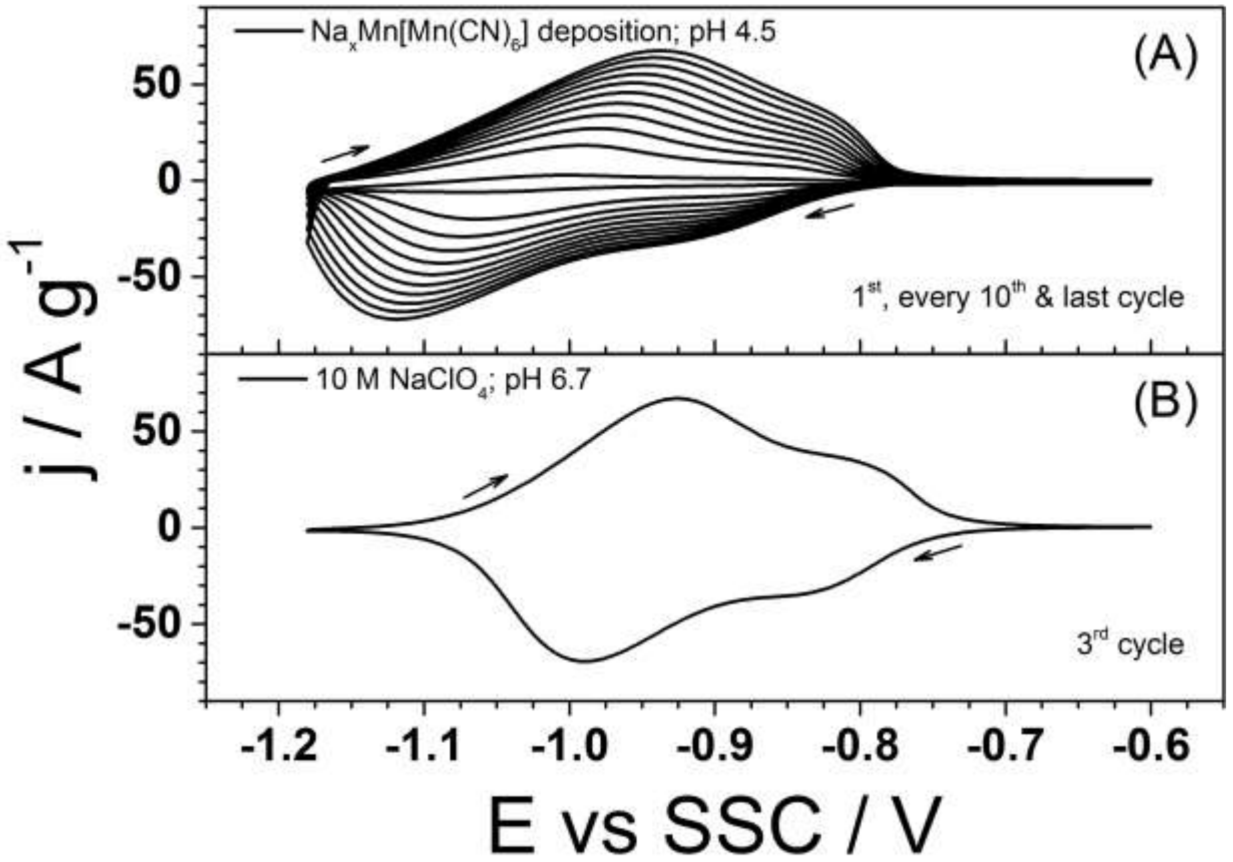

Figure S2. (A) Typical cyclic voltammogram recorded during electrochemical deposition of $Na_xMn[Mn(CN)_6]$ thin film at pH=4.5. (B) Typical cyclic voltammogram of $Na_xMn[Mn(CN)_6]$ thin film electrode in a 10M $NaClO_4$ aqueous solution at pH=6.7.

X-ray photoelectron spectroscopy (XPS) was performed using a SPECS XPS spectrometer (SPECS, Germany). The X-ray beam was generated in a XR50 X-ray tube by using an Al anode (12kV, 200W) with photon energy of 1486.61 eV and beam focus of 1.4 mm x 4 mm on the sample. The kinetic energies of the electrons were detected by a semi-spherical electron energy analyzer (PHOIBOS 150 2D CCD), operating with a pass energy of 20 eV to ensure a high resolution of the spectra. Prior to the XPS measurements, the samples were vigorously rinsed with distilled water. Examples of the XPS data for the resulting Co-, Cu- and Ni-based thin films before and after Na-intercalation are shown in Figure S3. The XPS data analysis shows, that the deposition of these PBA thin films was successful. The Na 1s peak is significantly increased after intercalation and fits to the literature values, taking into account that no pure materials are analyzed but chemical compounds[2]. $Na_2Co[Fe(CN)_6]$ and $Na_2Ni[Fe(CN)_6]$ show a similar trend, where the shape of the

transition metal ion peaks is not affected by the intercalation / deintercalation procedure, but the Fe lines deviate from each other. For Fe, two double peaks are visible in the de-intercalated state, where the peak with the higher and lower binding energy within each double peak belongs to the oxidation state Fe^{3+} and Fe^{2+}, respectively. After intercalation, the oxidation state of iron is lowered to Fe^{2+} and therefore the intensity of the peak with the lower binding energy is significantly increased.

However, $Na_2Cu[Fe(CN)_6]$ shows a different behavior compared to $Na_2Co[Fe(CN)_6]$ and $Na_2Ni[Fe(CN)_6]$, since there is no pronounced peak for Na 1s during deintercalation and also no clear trend regarding the transition metal ion or iron. Therefore, it is possible that not only iron but also copper is contributing to the capacity by changing the oxidation state.

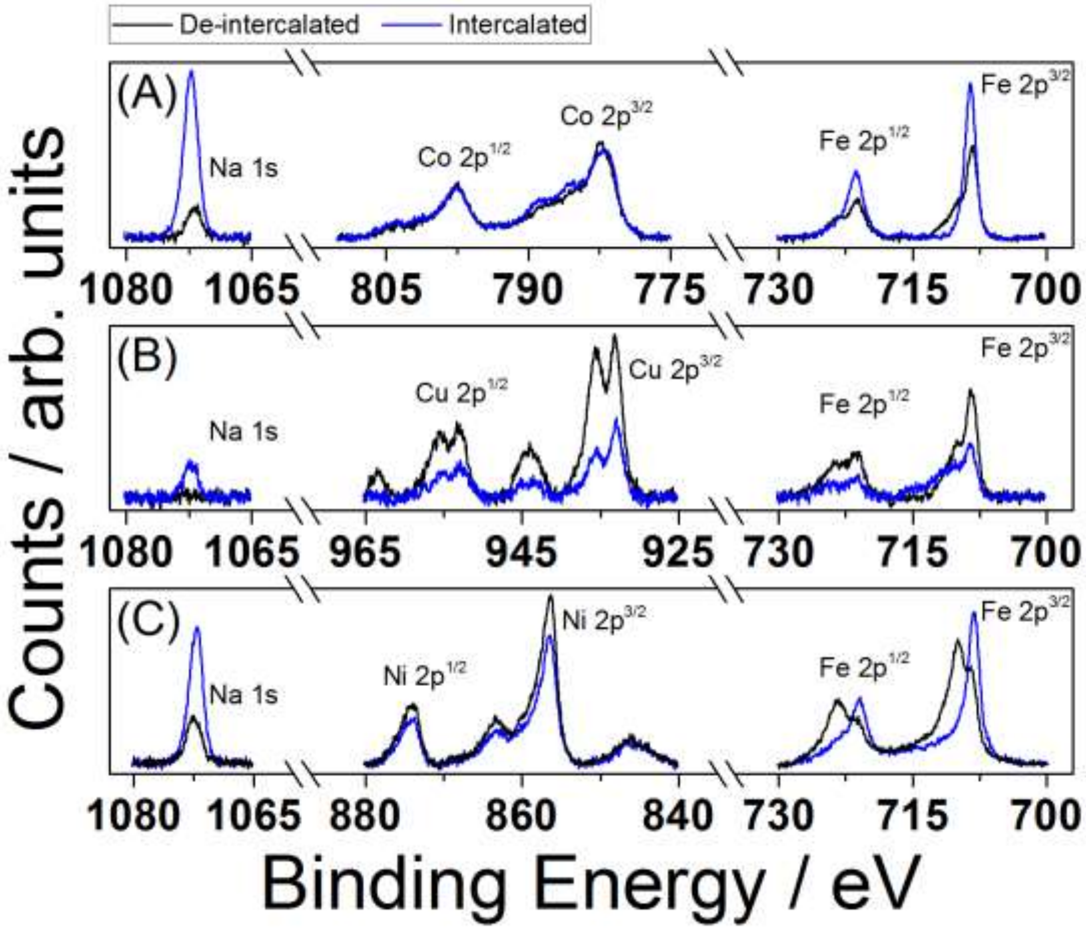

Figure S3. XPS for the deposited PBA thin films before (black curves) and after (blue curves) Na-intercalation. (A) The Co-containing PBA, (B) the Cu-containing PBA, and (C) the Ni-containing PBA thin films.

In case of the $Na_xMn[Mn(CN)_6]$ thin film (Figure S4) one can clearly see XPS peaks associated with Na and Mn. The presence of these elements in the sample, as well as further experimental analysis (AFM, Figure S5D; cyclic voltammetry, Figure S2B; EIS, Figure 2I) shows, that deposition of $Na_xMn[Mn(CN)_6]$ was successful.

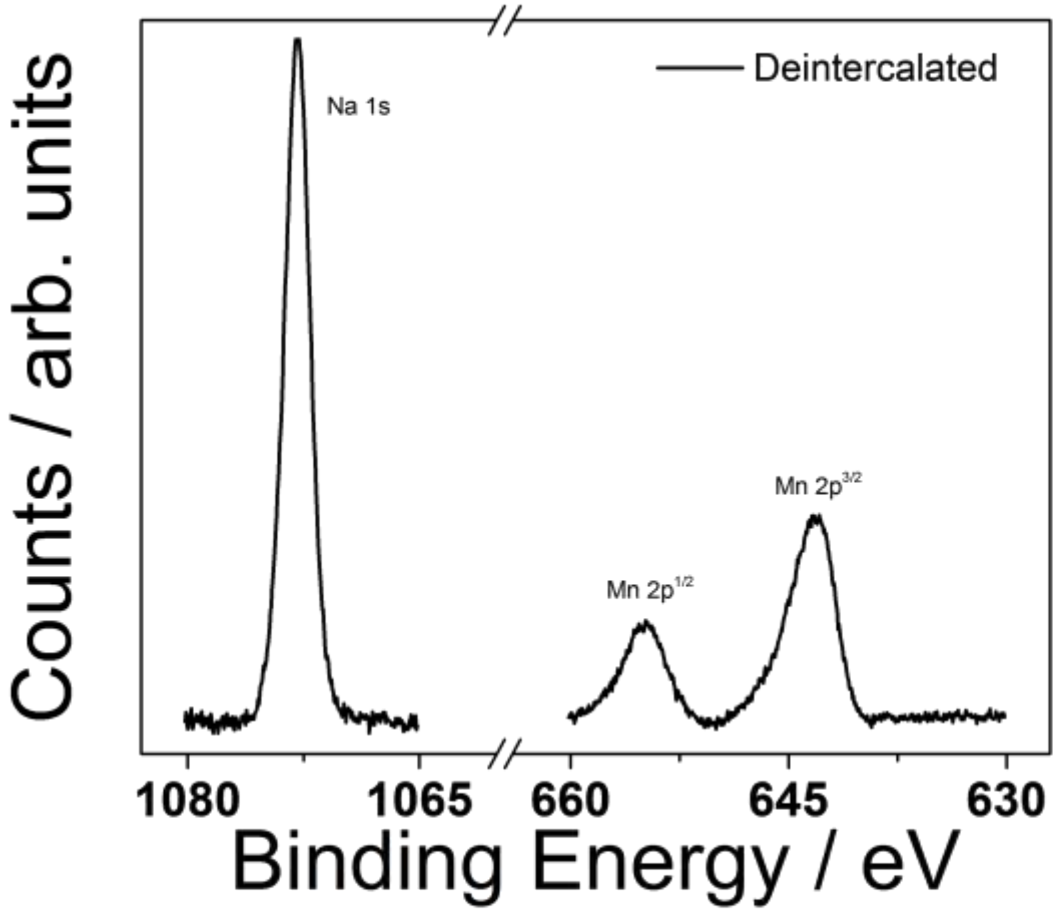

Figure S4. XPS for the deposited $Na_xMn[Mn(CN)_6]$ thin film before Na-intercalation.

AFM pictures of the resulting $Na_2Co[Fe(CN)_6]$, $Na_2Cu[Fe(CN)_6]$, $Na_2Ni[Fe(CN)_6]$ and $Na_xMn[Mn(CN)_6]$ thin films are shown in Figure S5. The atomic force microscope used to acquire the images was a multimode EC-STM/AFM instrument (Veeco VI) with a Nanoscope IIID controller using the Nanoscope 5.31r1 software. All measurements were conducted in tapping mode (AFM-tips BRUKER RTESP-300).

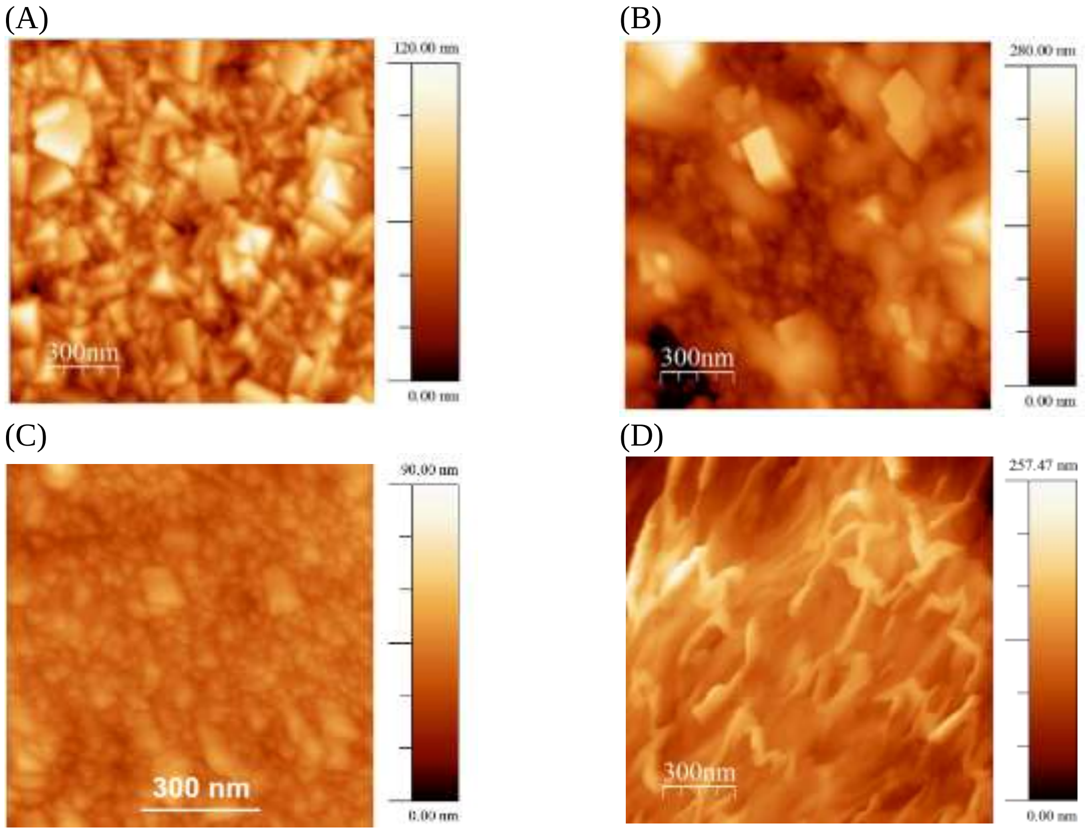

Figure S5. AFM characterization of (A) $Na_2Co[Fe(CN)_6]$, (B) $Na_2Cu[Fe(CN)_6]$, (C) $Na_2Ni[Fe(CN)_6]$ and (D) $Na_xMn[Mn(CN)_6]$ thin films.

It is noteworthy that the substrates are covered uniformly by particulate structures. However, the PBA thin film systems show differences in the basic morphology of these particulates. While Co-, Cu- and Ni-based thin films show rather polycrystalline structure with pyramidal, rectangular or roundish substructure, respectively, the Mn-based sample appears "flakey". Therefore, the samples with similar EIS response have also different morphology, suggesting that the impedance response is not influenced by the sample surface issues. Differences in the scales of the height profiles seem to be owed to singular, local deviations. This is, the $Na_2Cu[Fe(CN)_6]$ has a surface skewness of 0.49 while its RMS roughness equals 41.0 nm at an average profile height of 102.92 nm. The analysis of the $Na_xMn[Mn(CN)_6]$ thin film yields values of -0.15 for the surface skewness, 31.4 nm for the RMS roughness and 125.16 nm for the mean profile height.

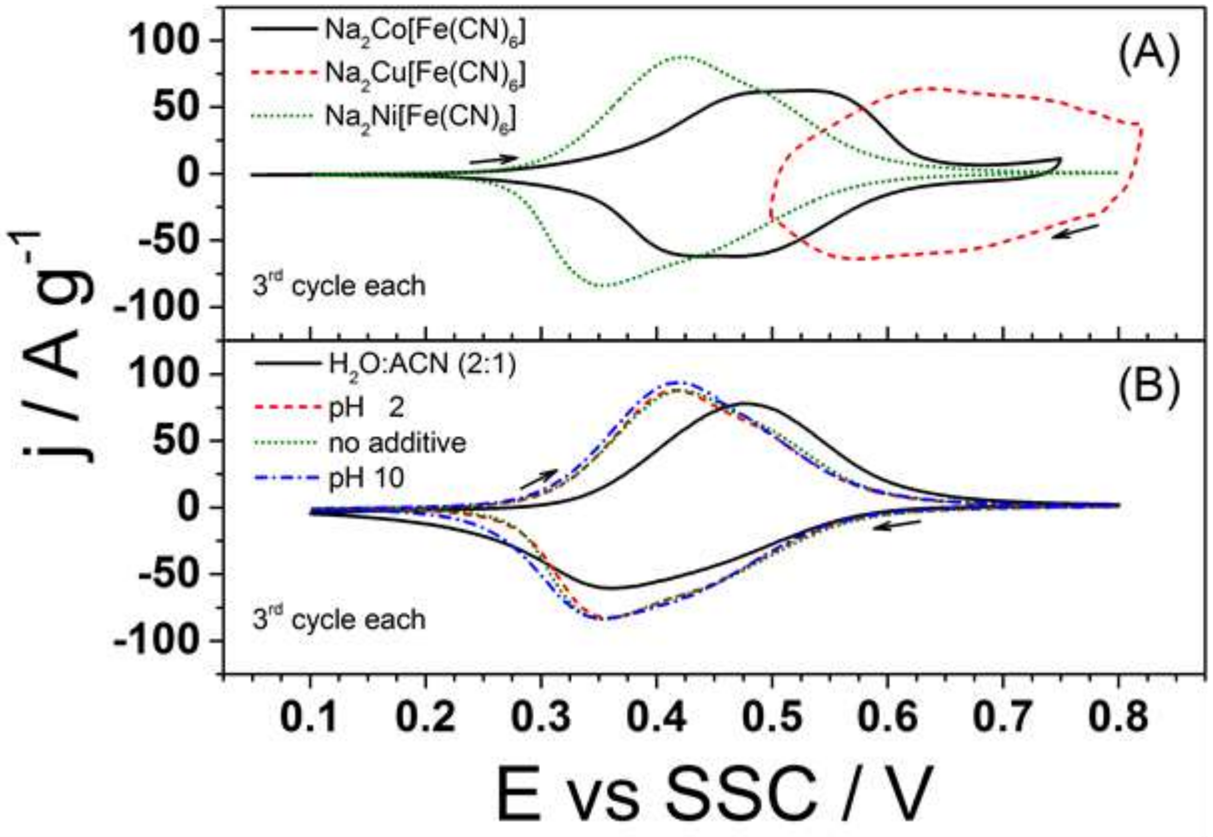

Figure S6. (A) Typical cyclic voltammograms obtained for the deposited PBA thin films in 0.25M Na_2SO_4 aqueous electrolytes, pH=7. (B) Typical cyclic voltammograms of $Na_2Ni[Fe(CN)_6]$ thin film electrodes in 0.25M Na_2SO_4 aqueous solutions at pH=2,7 and 10 as well as with the addition of ~33 vol.% of acetonitrile, ACN.

Na (de-)intercalation experiments (Figure S6) for each of these three thin film systems were performed in electrolyte containing 0.25 M Na_2SO_4. Additionally, $Na_2Ni[Fe(CN)_6]$ thin films were investigated in electrolytes containing 0.25 M Na_2SO_4 in H_2O:acetonitrile (2:1 volume ratio, 99.8 %, Sigma Aldrich) and 0.25 M Na_2SO_4 with pH=2 and pH=10 (Figure 4B). The pH of the latter was adjusted by adding H_2SO_4 or NaOH (>98 %, Sigma Aldrich) solutions, respectively. The experiments performed with the $Na_xMn[Mn(CN)_6]$ system (Figure S2B) employed an aqueous solution of 10 M $NaClO_4 \cdot H_2O$ (≥98.0%, Sigma Aldrich). All electrolytes were de-oxygenated with argon for > 15 minutes before usage. The potential ranges for (de-)intercalation were set as 0.05 V to 0.7 V vs SSC for $Na_2Co[Fe(CN)_6]$, 0.5 V to 0.82 V vs SSC for $Na_2Cu[Fe(CN)_6]$, 0.1 V to 0.8 V vs SSC for $Na_2Ni[Fe(CN)_6]$ and -1.18 V to -0.6 V vs SSC for $Na_xMn[Mn(CN)_6]$.

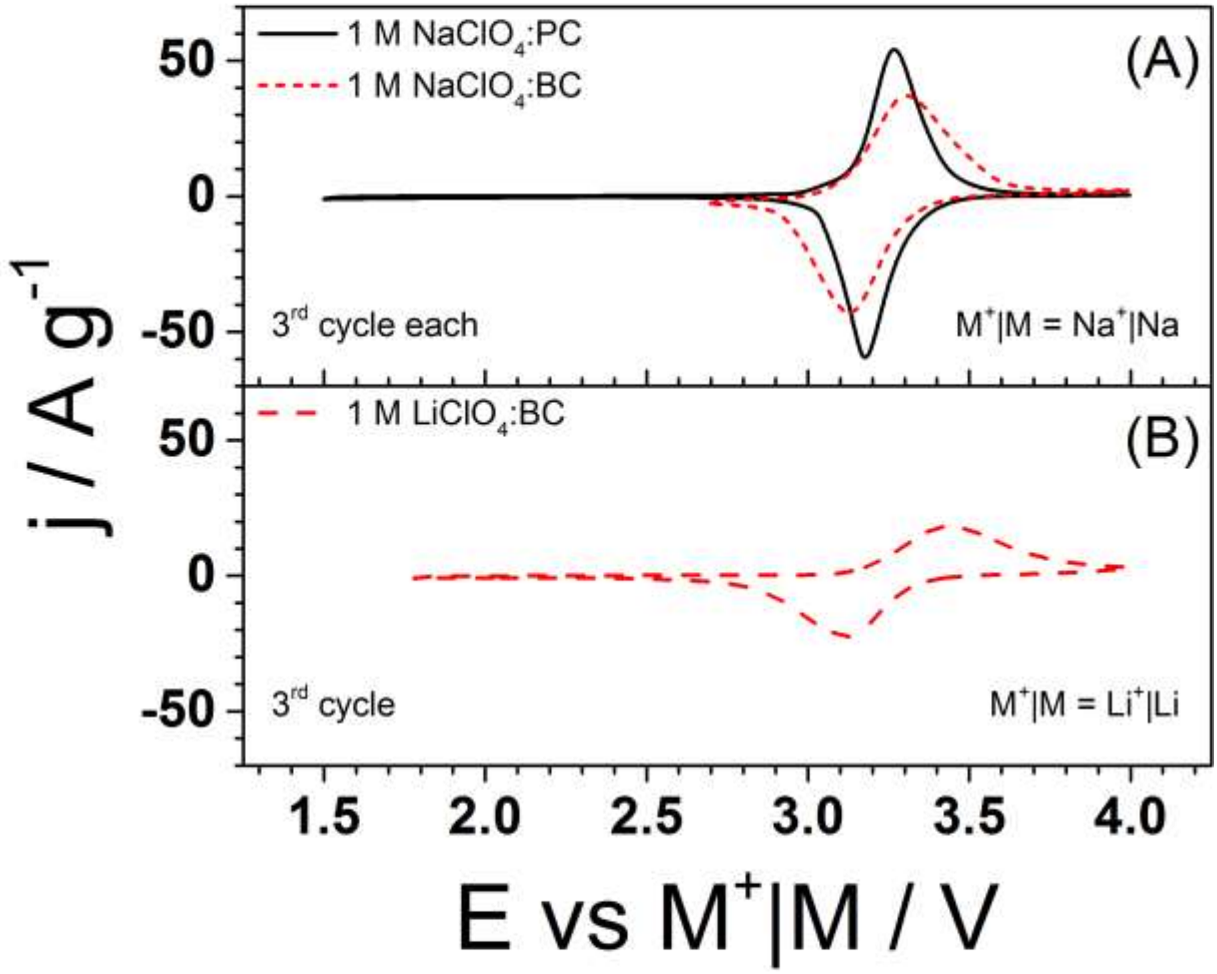

Figure S7. (A) Typical cyclic voltammograms of $Na_2Ni[Fe(CN)_6]$ thin film electrode in 1M $NaClO_4$ organic electrolytes employing propylene carbonate (PC) and 1,2-butylene carbonate (BC). The potential is referred to the electrochemical potential of the redox couple Na^+/Na. (B) Typical cyclic voltammograms of $Na_2Ni[Fe(CN)_6]$ thin film electrode in 1M $LiClO_4$ in BC. The potential is referred to the electrochemical potential of the redox couple Li^+/Li.

In addition to these aqueous systems, (de-)intercalation of Li^+- and Na^+-ions in Ni-based PBA thin films was also elucidated employing three organic electrolytes (Figure S7): 1 M $LiClO_4$ (99 %, anhydrous, packed under argon, Alfa Aesar) in 1 M $LiClO_4$ in 1,2-butylene carbonate (BC) (>98.0%, TCI), as well as 1 M $NaClO_4$ (98.0 %-102.0 %, anhydrous, Alfa Aesar) in propylene carbonate (PC) (99.7 %, anhydrous, Sigma Aldrich) and 1 M $NaClO_4$ in BC. In contrast to the investigations of $Na_2Ni[Fe(CN)_6]$ in aqueous systems, the thin films were deposited on an EQCM crystal plated with Au (SRS, Stanford, USA), still using the same protocol as described before. Experiments with these systems were performed in an argon-filled glove box (O_2 < 0.1 ppm and

H_2O < 0.1 ppm) by means of a cylindrical Teflon-cell. A receptacle which was shaped like a hollow cylinder and open on both bases was attached to the flat working electrode. The junction was sealed with a sealing ring and the resulting vessel filled with electrolyte. Two gold wires were coated either with metallic lithium or metallic sodium for systems with Li-salts or Na-salts, respectively, and immersed into the electrolyte as counter and reference electrodes. Potential ranges for cyclic voltammetry were chosen as 1.5 V to 4 V vs Na^+/Na for 1 M $NaClO_4$ in PC, 2.7 V to 4 V vs Na^+/Na for 1 M $NaClO_4$ in BC and 1.78 V to 4 V vs Li^+/Li for 1 M $LiClO_4$ in BC.

For further analysis of the thin films electrochemical impedance spectroscopy (EIS) using the same electrolytes as for intercalation experiments was carried out. To minimize artifacts caused by the potentiostat at high frequencies, a shunt capacitor (4.7 µF) was connected in parallel to the reference and counter electrode. The scan frequencies were set from 50 kHz to 100 mHz with 10 points per decade for aqueous systems and from 100 kHz to 100 mHz with 7 points per decade for investigation in organic electrolytes. A probing signal amplitude of 10 mV was used.

2. Preparation of TiO_2 and $Li_4Ti_5O_{12}$ electrodes

Commercially available $Li_4Ti_5O_{12}$ (2 m^2 g^{-1}) and anatase TiO_2 (100 m^2 g^{-1}) materials were received from MTI Corp (USA) and Sachtleben Chemie (Germany), respectively. Electrode slurries were prepared with 76:15:9 wt% composition of active material, C65 carbon black (Timcal) and polyvinylidene difluoride (PVdF) binder solution (Solef S5130, Solvay), respectively, dispersed in N-methyl pyrrolidone (NMP) (Sigma-Aldrich) and mixed thoroughly for 30 min at 4000 rpm using an ultra-turrax disperser (Ika). The slurry was then deposited onto a copper current collector using the “doctor blade” technique and dried at 60 °C overnight. 14 mm disk electrodes, with a 2.5 mm hole in the middle, were punched out with a commercially available hole punch (Hoffmann) and dried for 2 h at 105 °C in a vacuum oven (Büchi) resulting in an active material loading of *ca* 2 –

2.5 mg cm^{-2}. Cells were assembled in an argon-filled glove box (O_2 < 2 ppm and H_2O < 1 ppm). Metallic lithium (Sigma-Aldrich, d = 0.38 mm) was used as counter and reference electrodes, Whatman GF/D glass fiber filter as separator and 1 M $LiPF_6$ in ethylene carbonate (EC):diethyl carbonate (DEC), 1:1 wt% (LP40, Merck) as electrolyte. Scanning electron microscopy images for the $Li_4Ti_5O_{12}$ and anatase TiO_2 materials are shown in Figure S8.

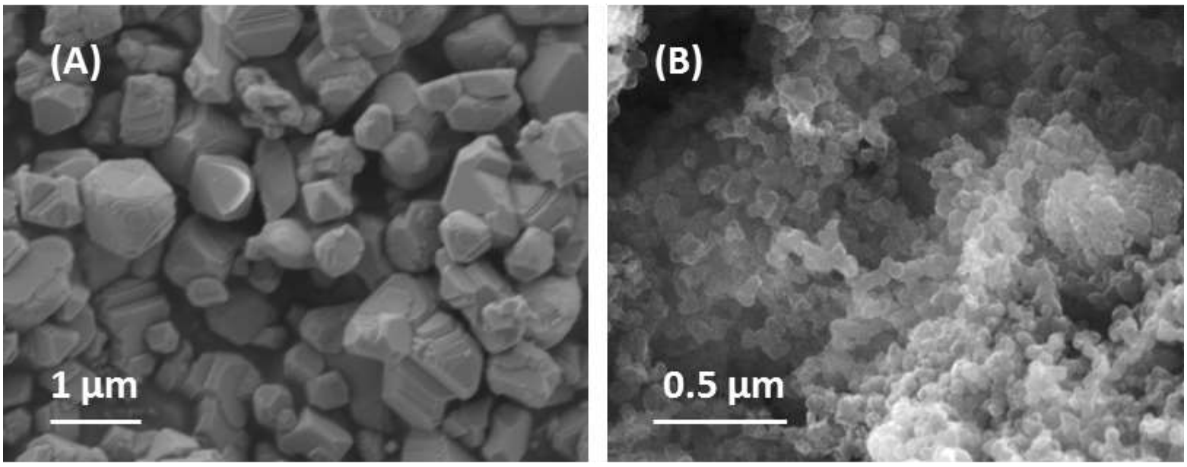

Figure S8. Scanning electron microscopy images of (A) $Li_4Ti_5O_{12}$ and (B) anatase TiO_2.

All electrochemical measurements in organic electrolyte solutions were performed using a three-electrode coaxial cell (Figure S9), which was shown to deliver highly reliable EIS data.[3-5] Moving the reference electrode to a coaxial position in combination with a precise alignment of the electrode stack optimizes the geometry of current lines, leading to reliable impedance spectra up to frequencies of 50 kHz. Compared to common three-electrode Swagelok cells, the coaxial configuration allows more precise EIS measurements.

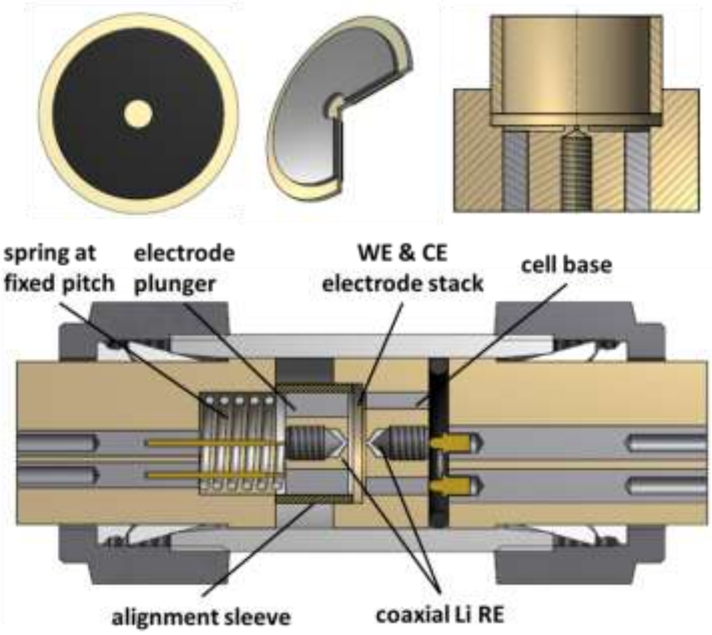

Figure S9. Improved coaxial cell with precise electrode alignment and defined spring pressure, suitable for thin separators like Celgard®; fitted to ¾ in. Swagelok® cells. Reproduced with permission from ref.[3] © 2012 Elsevier.

3. Theoretical EIS considerations

As a first approximation we propose the following general three-stage mechanism capable to explain the impedance data (all three steps are quasi-reversible):

(1) An electroactive step, i.e. very fast reversible oxidation/reduction of the transition metal (Figure S10), TM. AM - alkali metal cation, M' an "optional" transition metal cation which does not participate in the redox reactions, L_y is a ligand, e.g. CN^- or O^{2-}):

$$AM_xM'[TM^{m+}L_y] - 1e- \leftrightarrow AM_xM'[TM^{n+}L_y]^+$$

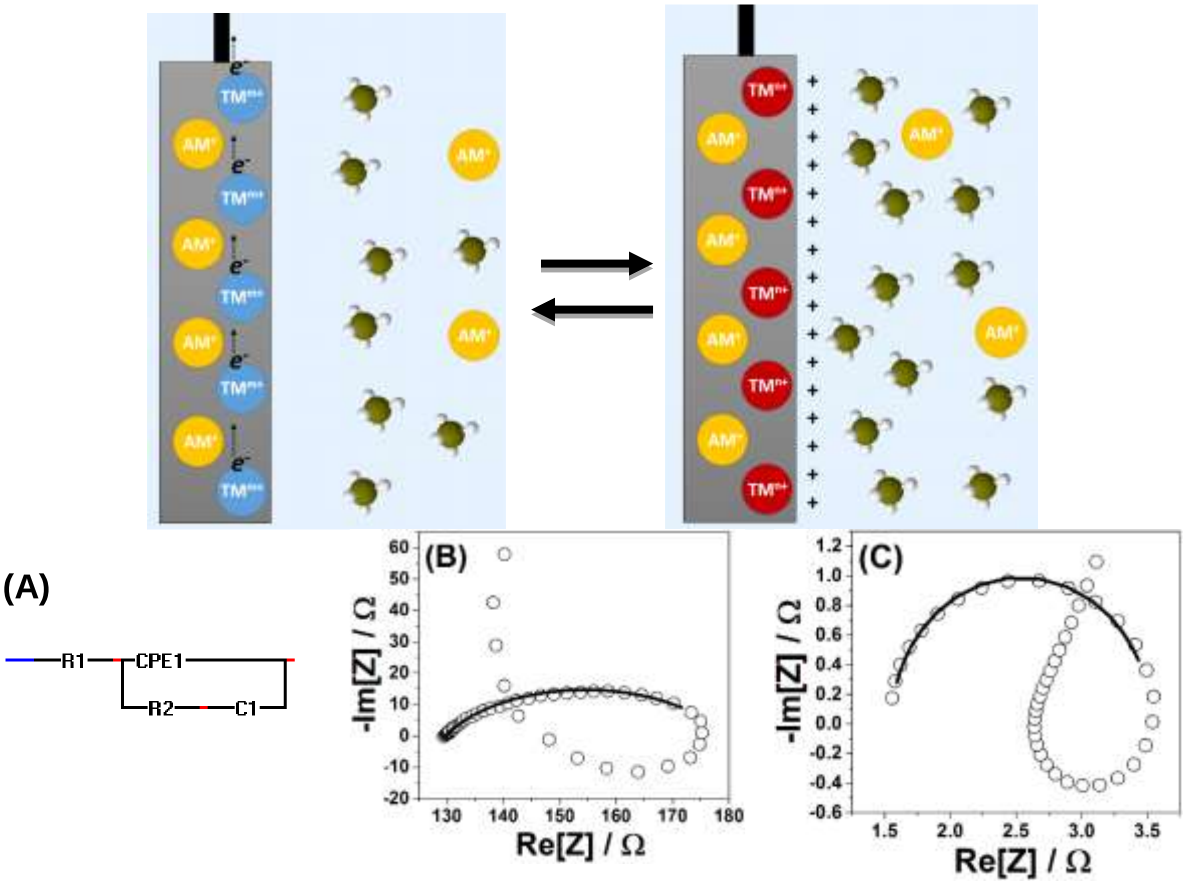

Figure S10. Schematic representation of the first reversible stage of the alkali metal cation intercalation/deintercalation. (A) "Classical" equivalent electric circuit in the case of a fast reversible one-stage surface limited redox reaction at the electrode surface. (B) The high frequency part of the spectrum for the $Na_2Ni[Fe(CN)_6]$ electrode in the aqueous media can be fitted with the equivalent circuit shown in (A). (C) The high frequency part of the spectrum for the TiO_2 electrode in the organic electrolyte can also be fitted using the equivalent circuit shown in (A). Designations: **AM^+ (yellow)** - alkali metal cations, **TM^{m+} (blue)** - transition metal cations in a reduced form, **TM^{n+} (red)** - transition metal cations in an oxidized form, green/white structures - anions.

The fast first stage manifests itself at high frequencies and can be described by the "classical" physical model shown in Figure S10A with very big values of pseudo-capacitances, C1. The examples of the fitting of the high frequency parts of the spectra are shown in Figure S10B,C.

(2) Specific adsorption of anions, e.g. SO_4^{2-}, which can be considered as a temporary compensation of the excessive positive electrode charge due to relatively slow AM^+ de-intercalation (the mass transport in solids is relatively slow comparatively with liquids):

$$2AM_xM'[TM^{n+}L_y]^+ + SO_4^{2-} \leftrightarrow (AM_xM'[TM^{n+}L_y])_2SO_4$$

The slower second stage is mutually interconnected with the first stage and manifests itself at lower frequencies (Figure S11). These frequency parts of the spectra can be described by the physical model (two-stage mechanism with a reversible specific adsorption) shown in Figure S11A where C1 and R3 can be formally positive or negative depending on actual values of the kinetic constants. The examples of the fitting of the high and middle frequency parts of the spectra are shown in Figure S11B,C. Notably, the model presented in Figure S10A cannot describe the low frequency parts of the spectra. Further general theoretical details regarding these partial models can be found in ref. [6]

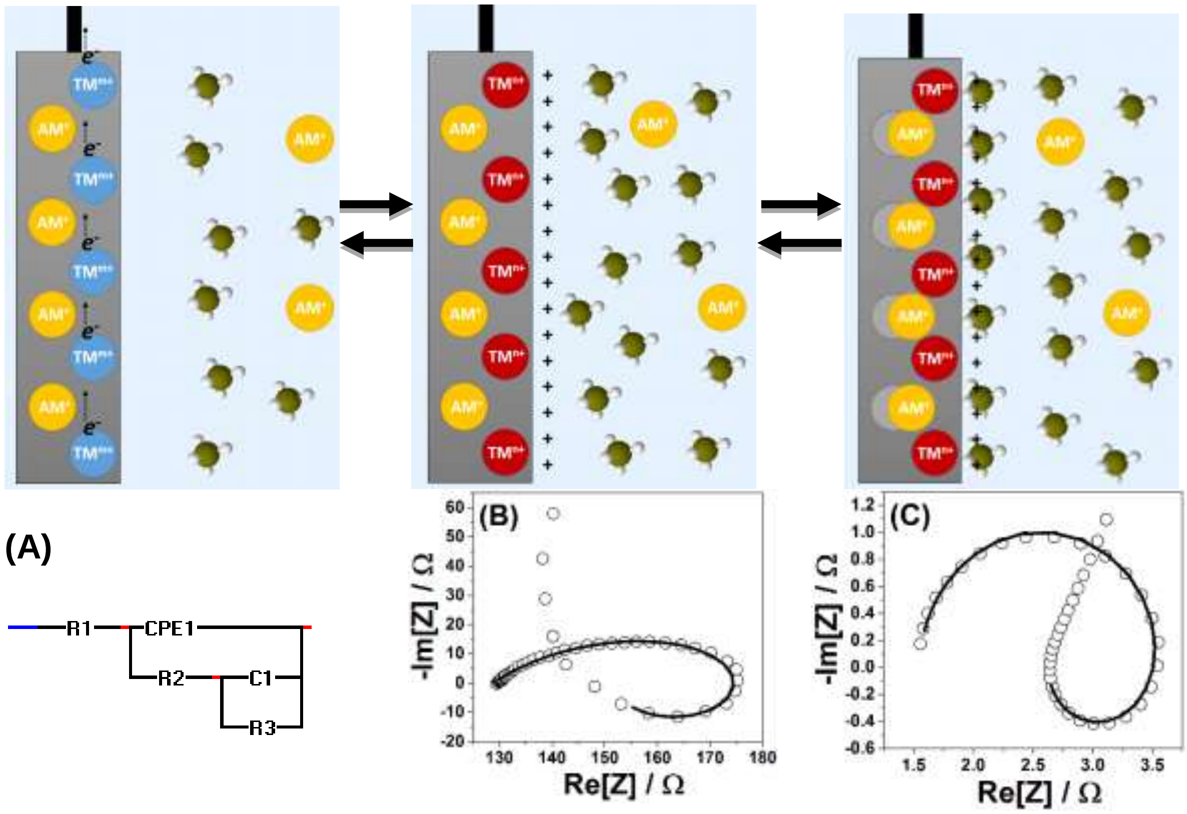

Figure S11. Schematic representation of the first (faster) and the second (slower) interconnected reversible stages of the alkali metal cation (de-)intercalation. (A) An equivalent electric circuit, where C1 and R3 can formally be positive or negative, depending on the values of the kinetic parameters. (B) The high and middle frequency parts of the spectrum for the $Na_2Ni[Fe(CN)_6]$ electrode in the aqueous media can be fitted with the equivalent circuit shown in (A). (C) The high and middle frequency parts of the spectrum for the TiO_2 electrode in the organic electrolyte can also be fitted using the equivalent circuit shown in (A). Designations: **AM^+ (yellow)** - alkali metal cations, **TM^{m+} (blue)** - transition metal cations in a reduced form, **TM^{n+} (red)** - transition metal cations in an oxidized form, green/white structures - anions.

(3) The “non-electroactive step” (no net interfacial charge transfer), AM^+ and anions leave the interface:

$$(AM_xM'[TM^{n+}L_y])_2SO_4 \leftrightarrow 2AM^+ + SO_4^{2-} + 2AM_{x-1}M'[TM^{n+}L_y]$$

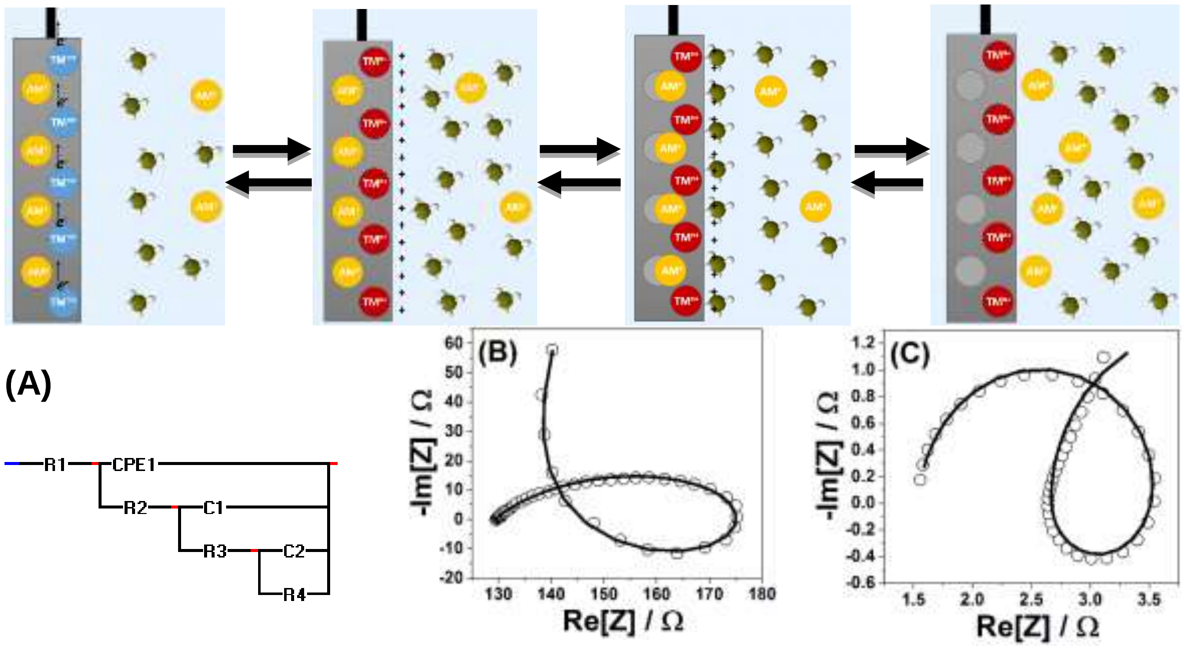

Figure S12. Schematic representation of the first (faster), the second (slower) and the third (even slower) interconnected reversible stages of the alkali metal cation intercalation/deintercalation (see SI text). (A) An equivalent electric circuit, where C1, C2, R3 and R4 can formally be positive or negative, depending on the values of the corresponding kinetic parameters. (B,C) Both the spectra for the PBA and Ti-based materials in aqueous and organic media, correspondingly, can be fitted using the same physical model shown in (A). Designations: **AM^+ (yellow)** - alkali metal cations, **TM^{m+} (blue)** - transition metal cations in a reduced form, **TM^{n+} (red)** - transition metal cations in an oxidized form, green/white structures - anions.

The even slower third stage is mutually interconnected with the first and the second stages and manifests itself at low frequencies (Figure S12). The spectra can be described by the physical model shown in Figure S12A where C1, C2 and R3, R4 can be formally positive or negative depending on actual values of the kinetic constants. The examples of the fitting are shown in Figure S12B,C. Fitting parameters of two further systems (Table S1) that are covered in the manuscript ($Na_2Ni[Fe(CN)_6]$ in 1 M $NaClO_4$ in PC (Figure 2G) and C-$LiFePO_4$ in 1 M $LiPF_6$ in EC:DEC (Figure 3F)) complement these. Notably, this is the simplest physical model capable to describe the spectra. Further general theoretical details regarding this model can be found in ref. [6]

Table S1. Examples for fitting parameters of the equivalent electric circuit used in this work (Figure 1); R_u a) - uncompensated resistance, Z_{dl} b) - impedance of the double layer, R_{ct} - charge transfer resistance, other R and C elements are adsorption (pseudo)resistances and (pseudo)capacitances, respectively). Listed are results for $Na_2Ni[Fe(CN)_6]$ electrode (1 M $NaClO_4$ in PC, Figure 2G) and C-$LiFePO_4$ electrode (1 M $LiPF_6$ in EC:DEC, Figure 3F).

System	R_u a), c) [Ω]	$\|Z_{dl}\|$ b) [µF]	n_{dl} b)	R_{ct} c) [Ω]	$R_{a,1}$ c) [Ω]	$R_{a,2}$ c) [Ω]	$C_{a,1}$ c) [µF]	$C_{a,2}$ c) [mF]
$Na_2Ni[Fe(CN)_6]$ (1 M $NaClO_4$ in PC)	125.18	6.53	0.61	373.86	-351.4	1348.8	-91.07	4.03
C-$LiFePO_4$ (1 M $LiPF_6$ in EC:DEC)	20.10	0.42	0.94	30.59	-4.49	20.81	-292.35	4.47

a) EIS spectra in all figures have been corrected for R_u; b) Z_{dl} is modeled as a constant phase element (CPE, Figures S10 – S12); c) R_u, R_{ct}, $R_{a,1}$, $R_{a,2}$, $C_{a,1}$ and $C_{a,2}$ in Figure 1 in the manuscript equal R_1, R_2, R_3, R_4, C_1 and C_2 in Figures S10 – S12 in the SI, respectively.

4. EIS measurements in organic electrolyte solutions

The phase transformation occurring during Li^+ (de-)intercalation in $Li_4Ti_5O_{12}$ and TiO_2 results in the appearance of a flat potential plateau in the potential profiles during the galvanostatic (dis-)charge of these materials. Therefore, EIS spectra were recorded at different state of charge. Galvanostatic intermittent titration (GITT) was thus combined with electrochemical impedance spectroscopy (EIS). During the GITT experiments, the samples were cycled galvanostatically with C/2 rate (87 mA g^{-1} and 168 mA g^{-1} for $Li_4Ti_5O_{12}$ and TiO_2 respectively) within the potential range from 3 to 1 V vs. Li/Li^+ (1 M Li^+). The current was interrupted after every 17.4 mAh g^{-1} or 33.6 mAh g^{-1} (for $Li_4Ti_5O_{12}$ and TiO_2, respectively) during the intercalation, and the sample was allowed to relax for 30 min. After each relaxation period of 45 min impedance spectra were acquired preceded by 10 min of application of the fixed potential reached during the relaxation step. A potential perturbation with a 10 mV amplitude was applied in the frequency range from 50 kHz to 5 Hz.

Figure S13 shows the potential profiles recorded for $Li_4Ti_5O_{12}$ and TiO_2 electrodes. The red points represent the state of charge at which three EIS spectra were recorded. EIS spectra (Figure S14) corresponding to those red points revealed that the multi-stage mechanism is not specific to a certain state of charge. Detailed interpretation of the evolution of the EIS spectra upon the state of charge is beyond the scope of this study. In addition, the characteristic feature revealing the multi-stage mechanism was also observed at different cycles, namely 1st, 3rd and 10th cycles, as shown in Figure S15.

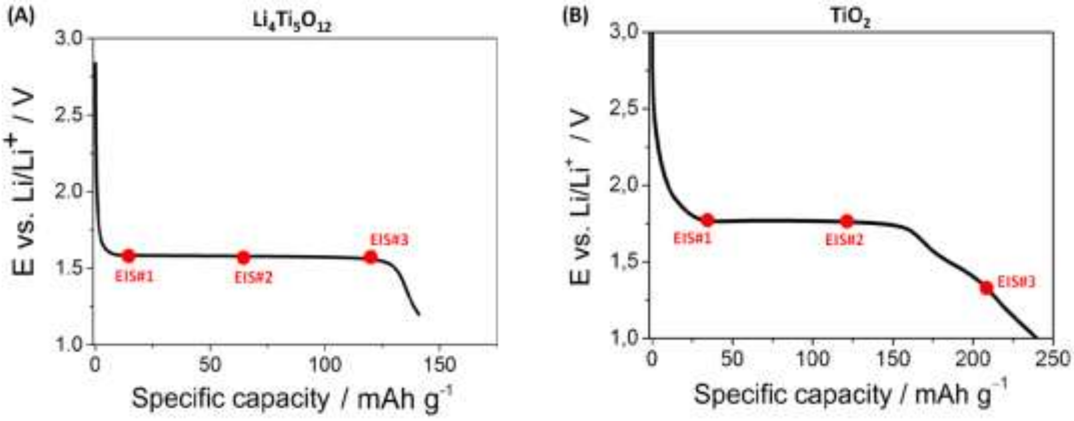

Figure S13. Potential profiles during the galvanostatic Li^+ intercalation in (A) $Li_4T_5O_{12}$ and (B) TiO_2. The red points represent three state of charge at which EIS spectra were recorded.

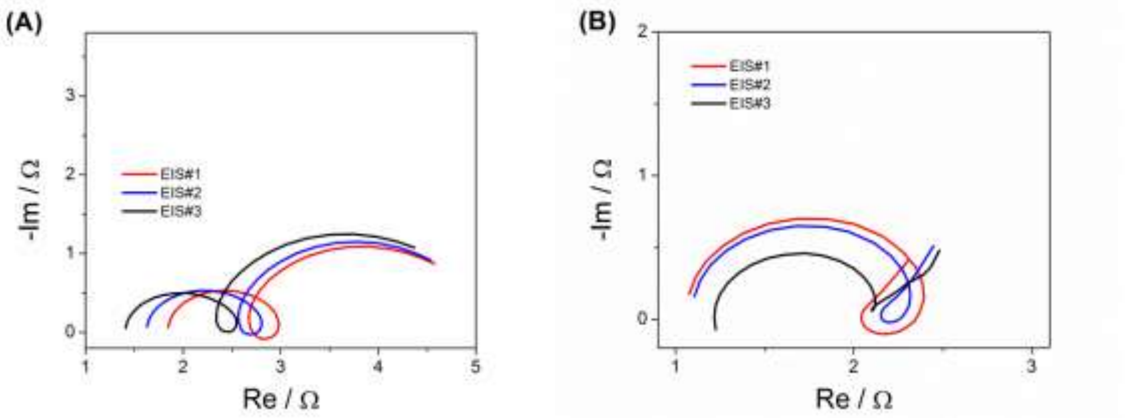

Figure S14. Evolution of EIS spectra upon the state of charge for the case of (A) $Li_4Ti_5O_{12}$ and (B) TiO_2. The three EIS spectra shown here correspond to the red points indicated in Figure S13.

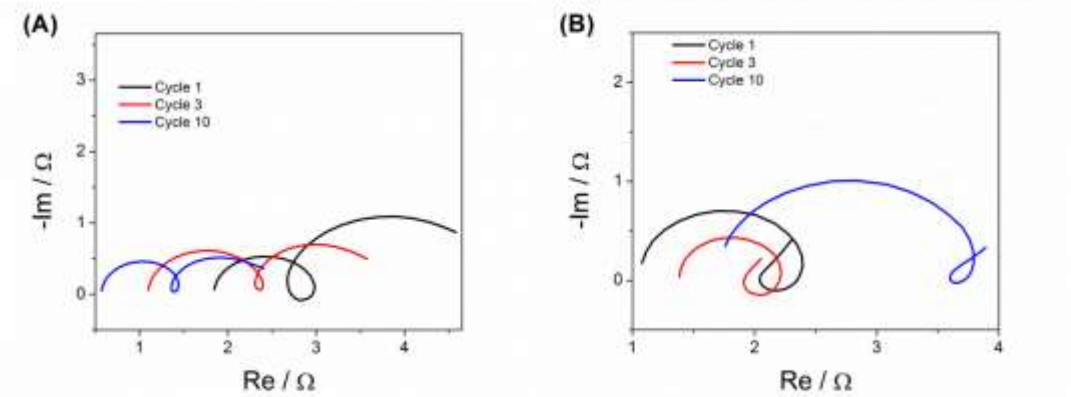

Figure S15. Evolution of EIS spectra (EIS#1) upon cycling for the case of (A) $Li_4Ti_5O_{12}$ and (B) TiO_2.

5. Examples of the results of the Krammers-Kronig check for EIS measurements

5.1. Aqueous electrolytes

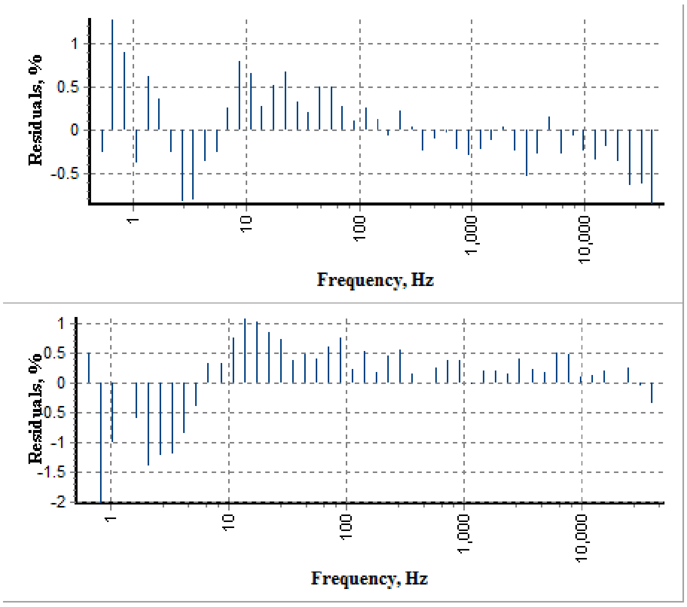

Figure S16. Examples of the results of the „logarithmic“ Krammers-Kronig check procedures for the impedance spectra obtained for $Na_2Ni[Fe(CN)_6]$ electrodes in aqueous 0.25M Na_2SO_4 electrolytes (pH=2).

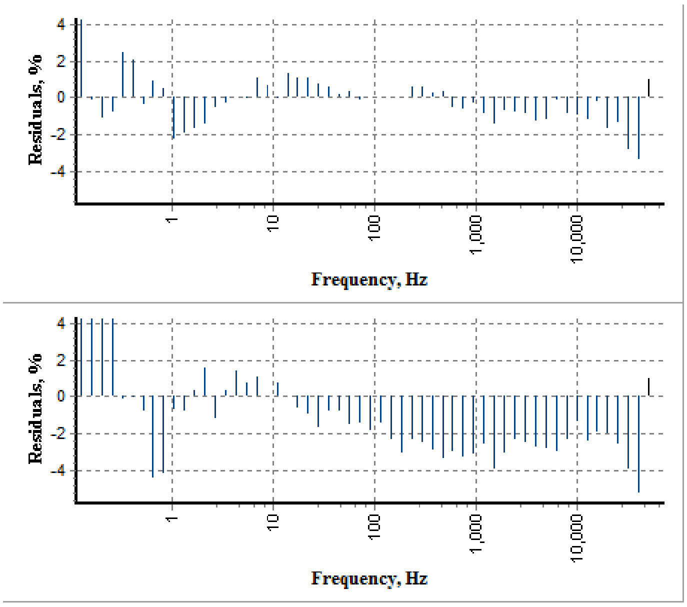

Figure S17. Examples of the results of the „logarithmic" Krammers-Kronig check procedures for the impedance spectra obtained for $Na_2Cu[Fe(CN)_6]$ electrodes in aqueous 0.25M Na_2SO_4 electrolytes, pH=7.

5.2. Organic electrolytes

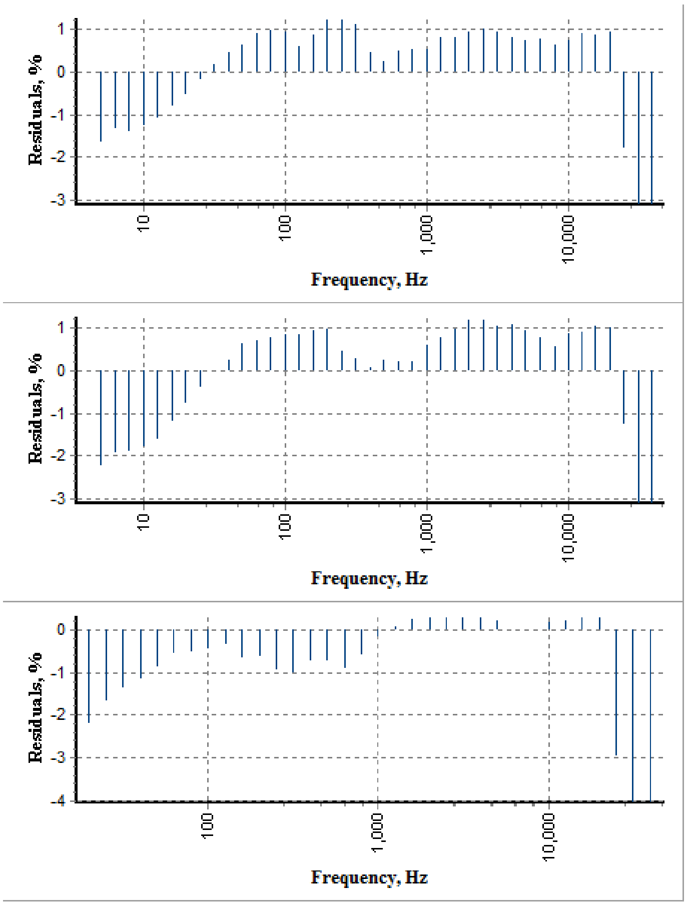

Figure S18. Examples of the results of the „logarithmic" Krammers-Kronig check procedures for the impedance spectra obtained for $Li_4Ti_5O_{12}$ electrodes in organic PC electrolytes.

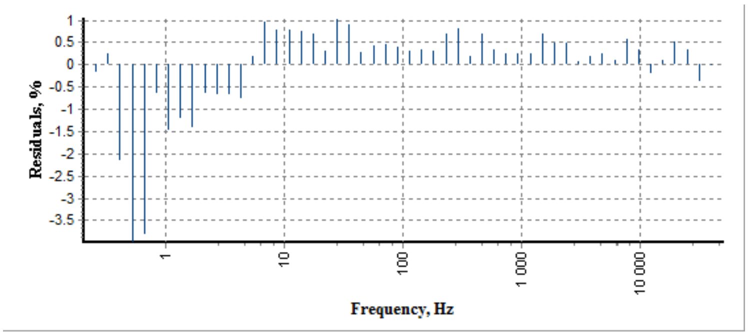

Figure S19. Examples of the results of „logarithmic“ Krammers-Kronig check procedures for the impedance spectra obtained for $Na_2Ni[Fe(CN)_6]$ electrodes in the 0.25M Na_2SO_4 electrolyte prepared using the mixture of H_2O and acetonytrile.

References

[1] G. Brauer, Handbook of preparative inorganic chemistry. Academic Press, New York, **1963**, 1474p.

[2] Thermo Scientific; XPS and Auger Handbook, System Manuals, Issue 2 (04/03)

[3] S. Klink, E. Madej, E. Ventosa, A. Lindner, W. Schuhmann, F. La Mantia, *Electrochem.Commun.* **2012**, *22*, 120.

[4] S. Klink, D. Höche, F. La Mantia, W. Schuhmann, *J. Power Sources* **2013**, *240*, 273.

[5] E. Madej, S. Klink, W. Schuhmann, E. Ventosa, F. La Mantia, *J. Power Sources* **2015**, *297*, 140.

[6] A. Lasia. Electrochemical Impedance Spectroscopy and its Applications. Springer-Verlag New York, **2014**, 367p.
